# Associations of neighborhood deprivation and household income during pregnancy on child externalizing and internalizing problems

**DOI:** 10.1017/S0954579426101151

**Published:** 2026-02-06

**Authors:** Yunzhe Hu, Julianna I Collazo Vargas, Christine Hockett, Katherine Ziegler, Natalie H. Brito, Anahid Akbaryan, Lauren A. Costello, Amy J. Elliott, William P. Fifer, Santiago Morales, Lauren C. Shuffrey

**Affiliations:** 1 Department of Psychiatry, Columbia University Irving Medical Center, New York City, NY, USA; 2 Department of Child and Adolescent Psychiatry, NYU Grossman School of Medicine, New York City, NY, USA; 3 Avera Research Institute, Sioux Falls, SD, USA; 4 Department of Applied Psychology, New York University, New York City, NY, USA; 5 Departments of Psychology and Pediatrics, Developmental and Brain and Cognitive Science Areas, University of Southern California, Los Angeles, CA, USA

**Keywords:** Child behavior problems, externalizing, household income, internalizing, neighborhood deprivation, socioeconomic status

## Abstract

Socioeconomic disadvantage has been established as a key risk factor for adverse child behavioral outcomes. Understanding how individual components of socioeconomic status (SES) interact with each other can elucidate protective factors and inform interventions and policies to promote positive developmental outcomes. This study examined the interactive effects of prenatal household income and neighborhood deprivation on child externalizing and internalizing problems (*N* = 793; *M*_age_ = 8.37 years; 51.2% females; 81.5% White). Results revealed an interaction effect between prenatal household income levels and neighborhood deprivation on child externalizing problems. Higher neighborhood deprivation was associated with higher child externalizing outcomes only at lower household income levels per person. Although no interaction between household income and neighborhood deprivation on child internalizing problems was observed, lower household income levels were independently associated with higher child internalizing problems. These findings underscore how prenatal individual- and neighborhood-level SES factors interact to shape children’s behavioral outcomes across childhood.

## Introduction

Socioeconomic status (SES) is a multidimensional construct encompassing several objective indicators, including household income, educational attainment, and occupation. Although existing literature defines and operationalizes family SES in various ways, a recent meta-analysis found that low SES, quantified by family income, parental education, and receipt of public assistance, is associated with higher levels of childhood psychopathology (Peverill et al., 2021). Children from socioeconomically disadvantaged households face an elevated risk of developing both externalizing problems (such as aggression, antisocial behavior, hyperactivity, conduct, and attention issues) and internalizing problems (such as anxiety, depression, somatic symptoms, and social withdrawal) (Leventhal & Brooks-Gunn, 2000; Xue et al., 2005). Broader social determinants, such as neighborhood characteristics, are also strongly linked to behavioral outcomes in children and adolescents, particularly externalizing and internalizing problems (Duncan et al., 1994; Flouri et al., 2012; Goodnight et al., 2012; Herzberg & Smyser, 2024; Kalff et al., 2001; Reijneveld et al., 2010; Rivera & Doom, 2023). Examining both individual- and neighborhood-level SES components, and their interactive effects, is crucial for identifying protective factors and informing targeted interventions to support early and middle childhood development.

Children may be especially vulnerable to environmental factors during the prenatal period, which can significantly impact early neurobehavioral development. Under the Developmental Origins of Health and Disease (DOHaD) framework, the prenatal environment has been recognized as an important indicator of future offspring health (Barker, 1995; Doi et al., 2022; Monk et al., 2019). SES disparities throughout the prenatal period have been consistently associated with a spectrum of adverse developmental outcomes, including lower birth weight, increased risk of preterm birth, alterations in neonatal brain structure, and deficits in neurocognitive functioning (Blumenshine et al., 2010; Herzberg & Smyser, 2024; Morales et al., 2024; Parker et al., 1994; Spann et al., 2020). For example, infants born to mothers with lower SES have exhibited differences in brain regions involved in emotion regulation, attention, sensory processing, and language development (Spann et al., 2020). Additionally, higher maternal educational attainment during the perinatal period has been associated with improved language and executive functioning in children (Morales et al., 2024). Similarly, lower income-to-needs ratios and lower maternal education levels during this period have independently been linked to poorer executive functioning in first-grade children (Hackman et al., 2015). Emerging research also indicates that prenatal SES disparities are associated with reduced inhibitory control, altered neural activity, such as reduced theta power, and increased externalizing problems (Xu et al., 2024).

Neighborhood adversity characteristics encompass factors, namely housing quality, availability of quality schools and services, average socioeconomic status, and crime rates (Minh et al., 2017). Independent of individual family circumstances, neighborhood-level SES is strongly associated with children’s brain development and behavioral outcomes (Buthmann et al., 2024; Kulla et al., 2024; Leventhal & Brooks-Gunn, 2000). Developmental research has shown that children residing in low-SES neighborhoods experience elevated stress levels and reduced access to social and institutional resources that support healthy development (Leventhal & Brooks-Gunn, 2000; Schenck-Fontaine et al., 2020). Neighborhood disadvantage, characterized by social and environmental conditions, has been associated with nearly twice the odds of externalizing and internalizing behavior problems among children aged 6 to 17 years (Singh & Ghandour, 2012). Across numerous studies, neighborhood adversity has been shown to exert a stronger effect on the development of externalizing symptoms (Goodnight et al., 2012; Leventhal & Brooks-Gunn, 2000; Reijneveld et al., 2010; Rivera & Doom, 2023; Singh & Ghandour, 2012) and, to a lesser degree, internalizing symptoms (Assari, 2021; Singh & Ghandour, 2012; Xue et al., 2005). Structural neighborhood disadvantage, measured by indicators of concentrated poverty, immigrant concentration, and residential instability, also correlates with increased internalizing problems across the lifespan (Xue et al., 2005). Similarly, insurance type, neighborhood disadvantage, and social risk at birth are associated with increased externalizing symptoms by two years of age (Ramphal et al., 2020).

Factoring in the complex components of neighborhood adversity, researchers have begun utilizing area deprivation as a multidimensional evaluation of a region’s SES conditions (Maroko et al., 2016). The Area Deprivation Index (ADI) measure is comprised of 17 census variables that capture socioeconomic disadvantage based on income, education, household characteristics, and housing (Maroko et al., 2016). Findings from the Adolescent Brain Cognitive Development (ABCD) study demonstrate that greater neighborhood deprivation, as indexed by higher ADI national percentile rank, is linked to reduced activation of motivational neural systems during reward anticipation (Mullins et al., 2020). Furthermore, reduced activation of the striatum and pallidum during reward anticipation mediated the link between higher area deprivation and increased attention problems in children (Mullins et al., 2020). Neighborhood deprivation, as indexed by the ADI, has also been associated with higher levels of externalizing problems in pre-adolescents in models adjusted for child and family characteristics, including family-level SES (Beyer et al., 2024).

Despite evidence highlighting that the independent contributions of individual-level SES factors and neighborhood adversity may affect child behavioral outcomes (Assari, 2021; Goodnight et al., 2012; Leventhal & Brooks-Gunn, 2000; Xu et al., 2024), no studies, to our knowledge, have evaluated the interactive effects of individual-level SES and the ADI during the prenatal period on child externalizing and internalizing problems. Individual-level SES factors may exacerbate or buffer the effects of neighborhood-level SES factors. Children in higher-income households often have access to extracurricular activities, which have been found to ameliorate the risks associated with living in disadvantaged neighborhoods (Fite et al., 2011). Similarly, higher household income and parental education are typically associated with greater parental involvement, a factor shown to buffer the negative effects of living in a more disadvantaged neighborhood (Kingston et al., 2013). Although the moderating role of individual-level SES factors of neighborhood-level SES effects has not been examined, these prior studies emphasize the importance of identifying which children, and under which contexts, are most susceptible to neighborhood risk to inform targeted intervention and prevention efforts.

## Current study

Neighborhood adversity profoundly influences a child’s development, with its strongest observed effects during early childhood and late adolescence (Buthmann et al., 2024; Flouri et al., 2012; Goodnight et al., 2012; Kalff et al., 2001; Kulla et al., 2024; Reijneveld et al., 2010; Rivera & Doom, 2023). Children who experience neighborhood adversity are at a higher risk of developing externalizing and internalizing problems (Goodnight et al., 2012; Leventhal & Brooks-Gunn, 2000; Reijneveld et al., 2010; Rivera & Doom, 2023). This heightened risk is due to the accumulation of SES factors, such as poor housing, crime, and lack of material and social resources, that negatively impact their environment during critical developmental periods (Singh & Ghandour, 2012). Concurrently, research indicates that the prenatal experiences of SES disadvantages can influence birth and child outcomes (Ncube et al., 2016). Although neighborhood effects on early childhood development may depend on a family’s circumstances (Minh et al., 2017), their interaction with individual-level SES factors remains poorly understood in particularly across childhood. Current literature has not examined the impact of prenatal neighborhood adversity, operationalized by the ADI, on child behavioral problems. Thus, the present study examines the interaction between individual-level socioeconomic status and neighborhood adversity during the prenatal period in relation to children’s externalizing and internalizing problems in early and middle childhood. Given the novel application of the ADI in a prenatal context, we hypothesized that the interactive effects between prenatal individual-level SES and neighborhood adversity on both externalizing and internalizing would be exacerbated at low household income levels.

## Method

### Study design and participants

Participants were a subset of mother-child dyads originally enrolled in the NIH Safe Passage Study (Dukes et al., 2014) conducted by the Prenatal Alcohol and SIDS and Stillbirth (PASS) Network. The PASS Network is a multi-center study investigating the role of prenatal exposure in risk for sudden infant death syndrome (SIDS), stillbirth, and fetal alcohol spectrum disorders. Eligibility criteria for the Safe Passage study sites in the United States included the ability to provide informed consent in English, being 16 years of age or older, and gestational age between 6 weeks and 40 weeks at the time of consent based on the estimated delivery date.

The current study’s maternal participants were enrolled in Rapid City or Sioux Falls clinics in South Dakota, aiming to increase adequate representation of South Dakota’s population and geographic variability. Child participants were followed up through the PASS-Environmental Influences on Child Health Outcomes (ECHO) in the Northern Plains study. The ECHO Program is a consortium of pediatric cohort studies across the United States that includes data from a shared protocol, with the goal of examining the effects of early life exposures on health outcomes throughout childhood and adolescence (Gillman & Blaisdell, 2018).

For the current study, data were available from 857 children with a minimum of one SDQ administration between 5 and 13 years of age. Participants were excluded from the current study due to no prenatal address reported at the time of enrollment in the PASS study (*n* = 40), inability to derive the prenatal ADI due to a lack of geocoded census block group with Federal Information Processing System (FIPS) code (*n* = 1), inability to derive the prenatal ADI state rank from the FIPS code potentially due to a block group with low population, a high proportion of residents living in group quarters, or missing data from a core component variable (*n* = 12), or no information on household income per person (*n* = 11). A total of 793 children born between February 2008 and January 2015 were included in our final sample (Table 1). Ethical approval was obtained from the Health Research Ethics Committee of Avera Research Institute and the WCG Institutional Review Board (IRB) with a reliance agreement with NYU Grossman School of Medicine and the University of Southern California.

**Table 1.** Participant demographic information

	Overall (*N* = 793)
**Child age at administration**	
Mean (SD)	8.43 (2.17)
Median [Min, Max]	9.00 [5.00, 13.0]
**Child Sex**	
Male	387 (48.8%)
Female	406 (51.2%)
**Child Race**	
American Indian or Alaska Native	84 (10.6%)
White	646 (81.5%)
Other or unknown	63 (7.9%)
**ADI State Rank**	
Mean (SD)	3.55 (2.58)
Median [Min, Max]	3.00 [1.00, 10.0]
**Monthly household income per person**	
Mean (SD)	1170 (640)
Median [Min, Max]	1170 [62.5, 2500]
**Gross income**	
Mean (SD)	3310 (1410)
Median [Min, Max]	3500 [ < = 250, >5000]
**Maternal educational attainment**	
Some primary school to some high school	39 (4.9%)
Completed high school	81 (10.2%)
Some college	217 (27.4%)
Completed college	294 (37.1%)
Post graduate	162 (20.4%)

*Abbreviation:* SD = standard deviation.

## Measures

### Maternal and child sociodemographic variables

Sociodemographic information, including address, monthly household income per person, maternal educational attainment, child’s date of birth, child’s age at assessment, child-assigned sex at birth, and child’s age, was collected via medical record abstraction or maternal self-report during pregnancy.

### Prenatal address and area deprivation index (ADI)

**Figure 1.**
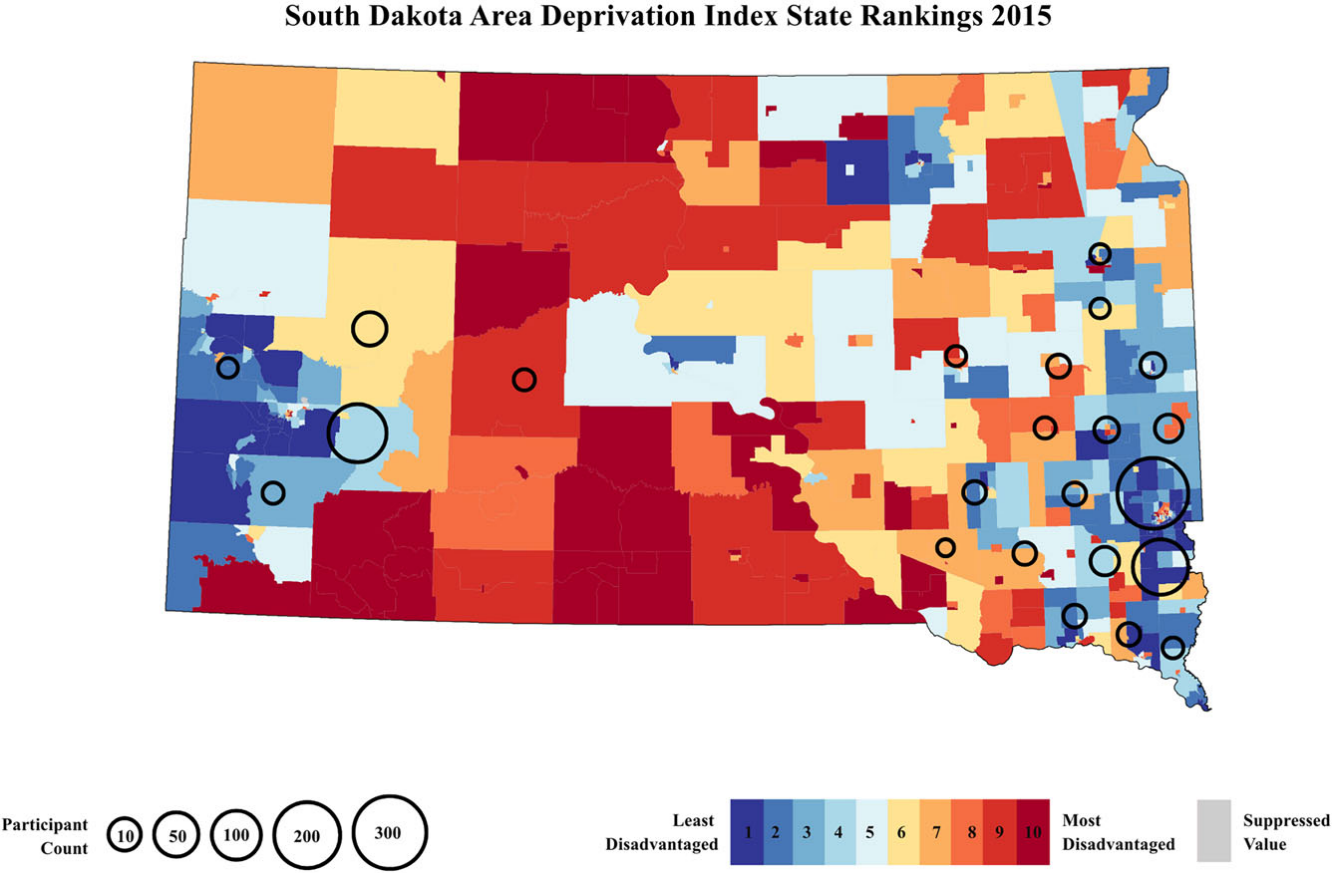
A heatmap of neighborhood deprivation state levels in South Dakota based on the 2015 area deprivation index state ranking. ADI state ranks range in deciles from 1 to 10, with decile 1 areas in dark blue representing the lowest level of neighborhood deprivation and decile 10 areas in dark red representing the highest level of neighborhood deprivation in South Dakota. Areas in gray represent suppressed values due to low and high group quarters populations. Areas with circles, ranging from 10–300 participant count, indicate locations with a high density of participants (*N* = 793).

Addresses were geocoded in geocod.io to generate 12-digit U.S. Census block group FIPS codes. Census block groups are geographical blocks for which the Census Bureau tabulates data and are delineated by the U.S. Census Bureau. The ADI is a ranking score of neighborhoods by socioeconomic disadvantage, composed of 17 education, employment, income, and housing quality measures from long-form census data. This composite measure is updated annually to incorporate data from the most recent American Community Survey (Kind & Buckingham, 2018). The ADI has been used extensively in research on associations between neighborhood disadvantage and social and health outcomes, including child cognitive functioning (Kalb et al., 2023), and neonatal intensive care unit mortality and morbidity (Sullivan et al., 2023). Neighborhoods, defined as census block groups and identified using FIPS codes, are assigned an ADI national and state rank. For this study, the ADI state rank was used since all participants resided in the state of South Dakota. ADI state ranks derived from the 2015 Version are calculated on a decile scale ranging from 1 to 10, with 10 representing the most disadvantaged decile in the state and 1 representing the least disadvantaged (Figure 1). To contextualize participants’ neighborhood deprivation within the broader U.S. landscape, we additionally included a heatmap of ADI national percentile ranks, which range from 1 to 100, highlighting South Dakota’s relative standing within the broader variability of neighborhood deprivation across the U.S. (see online supplement, Figure S1).

### Monthly household income per person

**Figure 2.**
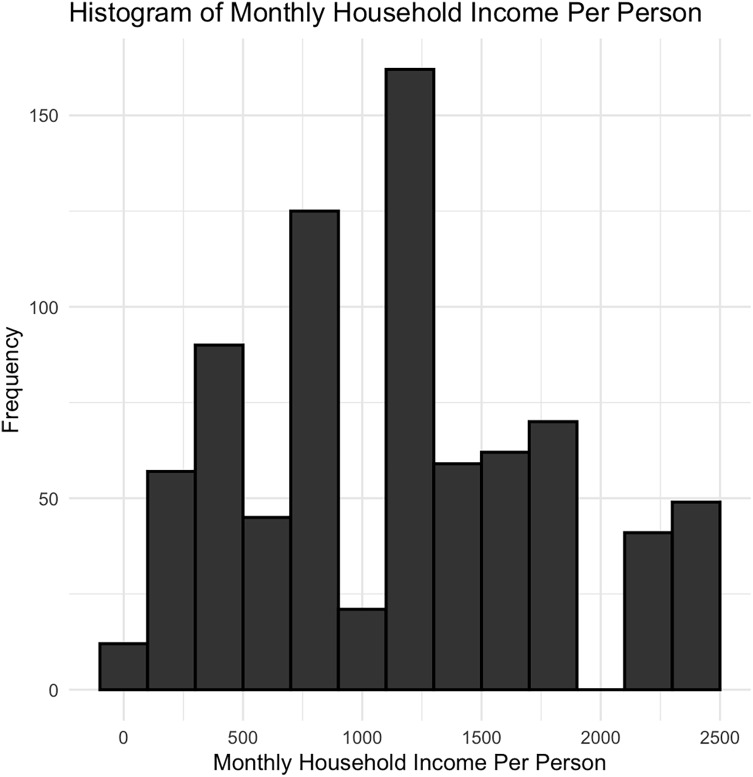
Histogram showing the distribution of monthly household income per person among participants (*N* = 793). Income values were grouped in intervals of $200. Most households report an income between $62.50 and $2,500 per person a month.

Household income per person was measured during pregnancy and obtained through participant self-report using the question “Including yourself and the number of people currently contributing, what is your current monthly household income (in US Dollars)”. Response options included: “Less than or equal to 500”, “501–1000”, “1001–2000”, “2001–3000”, “3001–4000”, “4001–5000”, “Greater than or equal to 5001” (Figure 2).

Total gross household monthly income was calculated based on the midpoint of an income range category, with midpoints of 250, 750, 1500, 2500, 3500, 4500, and >5000. Household income per person was then calculated by taking the total gross household monthly income divided by the total number of people supported by this income, obtained through participant self-report using the question “Including yourself, how many people are currently supported by this income: (specify number).”

### Maternal educational attainment

Maternal educational attainment was measured during pregnancy and obtained through participant self-report. Participants were asked to select the highest level of completed education from the following seven levels: “Some Primary School,” Completed Primary School,” Some High School,” Completed High School,” Some College,” Completed College,” and Post Graduate.” We collapsed the categories of Some Primary School,” Completed Primary School,” and Some High School,” due to small group sizes, and created a Some Primary School to Some High School” category.

## Child externalizing and internalizing behaviors

### Strengths and difficulties questionnaire (SDQ)

The SDQ is a brief parental-report questionnaire used to rate the behavior of children and adolescents on a three-point Likert scale. The questionnaire consists of 25 questions split up into five scales of emotional symptoms, conduct problems, hyperactivity/inattention, peer relationship problems, and prosocial behavior. The externalizing scale consists of the hyperactivity/inattention and conduct problem subscales. The internalizing scale consists of the emotional symptoms and peer symptoms subscales. The externalizing and internalizing scales have been suggested to be appropriate metrics in low-risk or general-population studies (Goodman et al., 2010). In a study of children aged 5–15 years, the SDQ demonstrated good psychometric properties, including internal consistency (α = 0.73), cross-informant correlation (*M* = 0.34), and test-retest reliability after 4 to 6 months (*M* = 0.62) (Goodman, 2001). In a follow-up analysis of the same sample, the SDQ scale showed adequate reliability across subscales (Xu et al., 2024). Within our sample, our internal consistency was good for the externalizing scale (α = 0.81) and hyperactivity/inattention subscale (α = 0.81), while adequate for the internalizing scale (α = 0.64) and conduct subscale (α = 0.60).

### Statistical analyses

All statistical analyses were conducted using R version 4.2.2 (Team, 2022). Descriptive statistics were used to summarize the characteristics of the study population, including child age, sex assigned at birth, maternal race, maternal educational attainment, and household income during pregnancy. We tested for differences in monthly household income per person, ADI state rank, maternal educational attainment, and age between children who were administered the SDQ once versus children who were administered the SDQ twice using Analysis of Variance (ANOVA). The correlation between demographic variables of sex, monthly household income per person, and education was calculated using point-biserial correlation for binary variables and Spearman correlation for calculations involving ordinal or continuous variables, which are provided in the online supplement.

Given the presence of repeated SDQ assessments for some participants (24.21%), primary analyses consisted of unadjusted and adjusted linear generalized estimating equation (GEE) models using the “geeglm” package (Højsgaard et al., 2005). The GEE models account for clustered observations (i.e., more than one SDQ assessment per individual) to examine the interactive and main effects of the ADI state rank and monthly household income per person on child externalizing and internalizing total scores on the SDQ. Adjusted models included the child’s age at assessment in years and the child’s assigned sex at birth as covariates.

Since externalizing symptoms encompass a broad and heterogeneous dimension of several disorders, *post hoc* exploratory analyses probed significant interactive effects within the externalizing domain by examining the hyperactivity and conduct problems subscales in adjusted models. These *post hoc* analyses can help determine whether the associations with prenatal individual-level SES and neighborhood disadvantage are across the externalizing spectrum or externalizing subscale-specific. This helps better understand the comorbidity across behavior problem dimensions, clarifies developmental pathways, and improves the interpretability and clinical relevance of our findings. *Post hoc* sensitivity analyses tested the robustness of the interaction with income by replicating models with maternal education as the predictor. Finally, to ensure that findings were not driven by site differences, we re-estimated the primary models within the Sioux Falls site as an additional sensitivity analysis, finding the same pattern of results. Results from the *post hoc* analyses and within-site sensitivity analyses are included in the online supplement.

For all models, we report the Wald χ^2^, unstandardized regression coefficients (*β*), and the standard error of estimates for each main effect or the significant interaction term.

## Results

### Sample characteristics

The final sample consisted of 793 children (Table 1). Maternal-child dyads were consented in either Rapid City (*n* = 208) or Sioux Falls (*n* = 585), South Dakota. A total of 192 (24.21%) children were administered the SDQ at two different time points, while all others (*n* = 601) were administered the SDQ at one single time point. We found no significant differences in prenatal monthly household income per person (*p* = 0.908), ADI state rank (*p* = 0.123), and maternal education (*p* = 0.145) between children who were administered the SDQ once versus children who were administered the SDQ twice. There was a significant difference (*p* = 0.018) in age between children who were administered the SDQ once (*M*age = 8.53) versus children who were administered the SDQ twice (*M*age = 8.14) (see online supplement, Table S1). The mean age at assessment across all administration points was 8.37 (*SD* = 2.19) years. A total of 387 (48.8%) children were assigned male at birth, and 406 (51.2%) children were assigned female at birth. Mothers self-reported their children to identify as American Indian or Alaskan Native (*N* = 84, 10.6%), White (*N* = 646, 81.5%), or Other or Unknown race (*N* = 63, 7.9%). The mean household monthly income per person during pregnancy across our sample was $1170 USD, (*SD* = 640). The mean monthly household gross income across our sample during pregnancy was $3310 USD, (*SD* = 1410). Mothers self-reported their educational attainment as some primary school to some high school (*N* = 39, 4.9%), completed high school (*N* = 81, 10.2%), some college (*N* = 217, 27.4%), completed college (*N* = 294, 37.1%), or completed a postgraduate degree (*N* = 162, 20.4%). There were significant site differences in both prenatal monthly household income per person (*p* < .001) and ADI state rank (*p* < .001), with participants recruited in Sioux Falls having higher monthly household income per person and lower ADI state rank. To account for these differences, we conducted sensitivity analyses within Sioux Falls, the site with the larger sample, to ensure that results were not driven by site differences. Importantly, these sensitivity analyses showed the same pattern of results as the ones reported below (see online supplement for details, Figures S2 and S3).

### Association of ADI state rank and monthly household income per person with child externalizing problems

**Figure 3.**
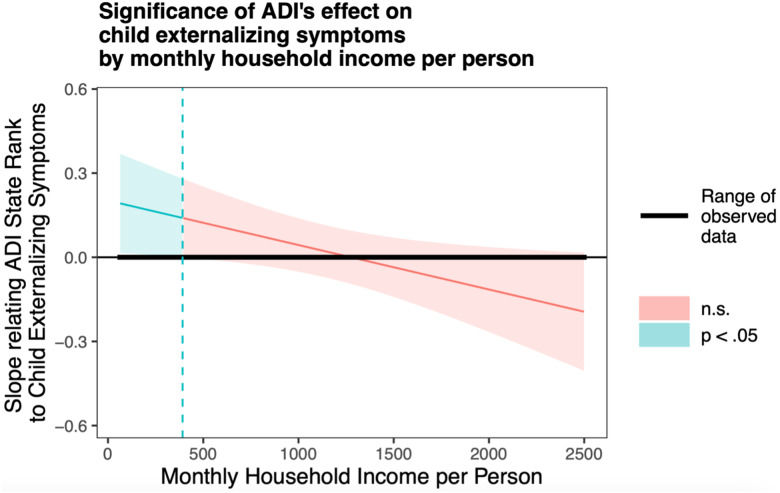
Probing unadjusted interactions with the Johnson-Neyman technique. The *y*-axis represents the conditional slope of ADI State Rank as the predictor, while the *x*-axis represents household income per person in US dollars. The plot shows where the conditional slope differs significantly from zero. Higher prenatal maternal ADI state rank was associated with higher child externalizing symptoms at low monthly household income per person levels, as indicated by the light blue area on the left, where *p* < 0.05.

**Figure 4.**
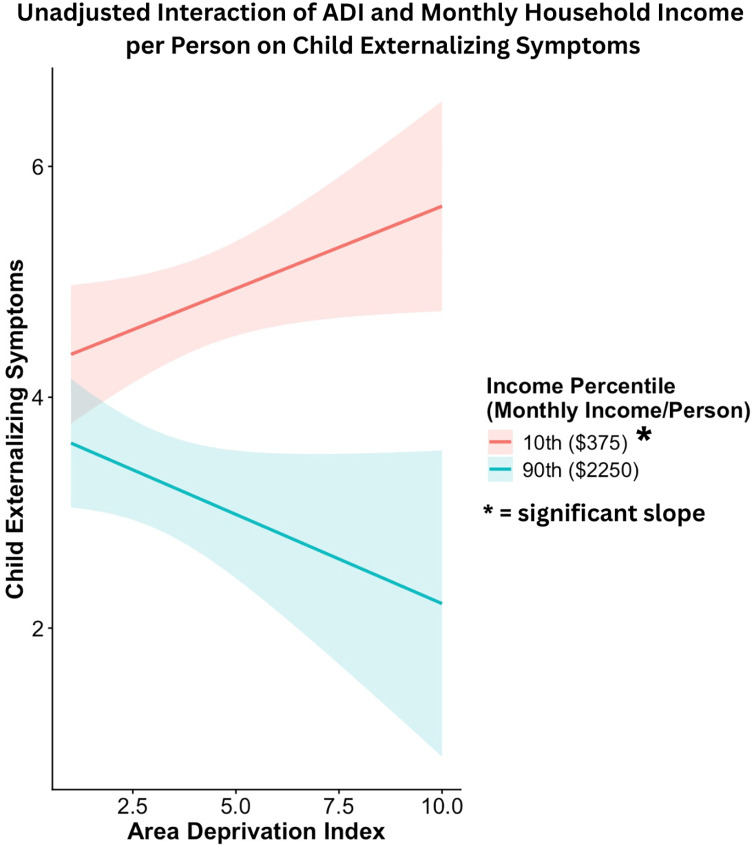
Simple slope analyses illustrating the interaction between ADI state rank and monthly household income in predicting child externalizing symptoms. Higher prenatal maternal ADI state rank was associated with higher child externalizing symptoms at low monthly household income per person levels.

Generalized linear models showed an interactive effect of ADI state rank and monthly household income per person on child externalizing problems in unadjusted (*β* = −0.076 ± 0.034, Wald χ^2^ = 4.99, *p* = .026) and adjusted models (*β* = −0.069 ± 0.032, Wald χ^2^ = 4.64, *p* = .031). As shown in Figures 3 and 4, a higher ADI state rank was associated with higher child externalizing problems only at lower levels of monthly household income per person. The association between ADI state rank and child externalizing problems was not significant at average or high levels of monthly household income per person. Significant covariates in the adjusted model included child age at assessment (*β* = −0.16 ± 0.032, Wald χ^2^ = 26.15, *p* < .001) and sex (*β* = −0.44 ± 0.067, Wald χ^2^ = 43.45, *p* < .001). Younger child age and male sex were each associated with higher child externalizing problems. Sensitivity analyses conducted within the Sioux Falls site showed the same pattern of results (see online supplement for details).

### Association of ADI state rank and monthly household income per person with child hyperactivity/inattention scores

**Figure 5.**
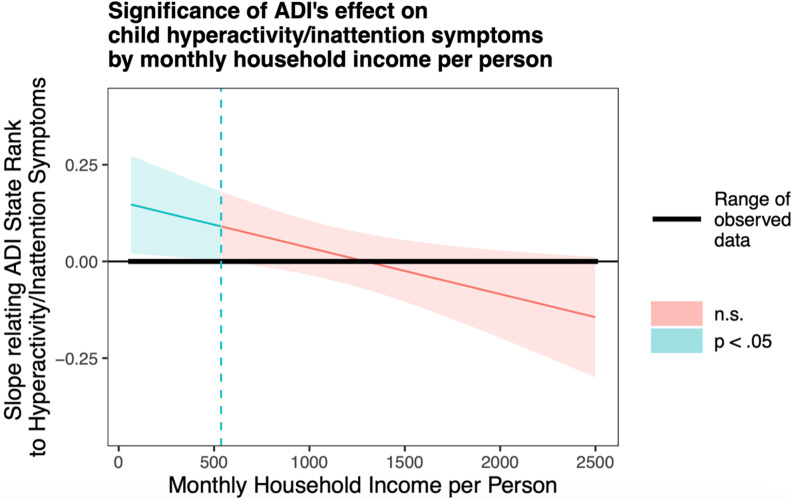
Probing unadjusted interactions with the Johnson-Neyman technique. The *y*-axis represents the conditional slope of ADI State Rank as the predictor, while the *x*-axis represents household income per person in US dollars. The plot shows where the conditional slope differs significantly from zero. Higher prenatal maternal ADI state rank was associated with higher child hyperactivity/inattention symptoms at low monthly household income per person levels, as indicated by the light blue area on the left, where *p* < 0.05.

**Figure 6.**
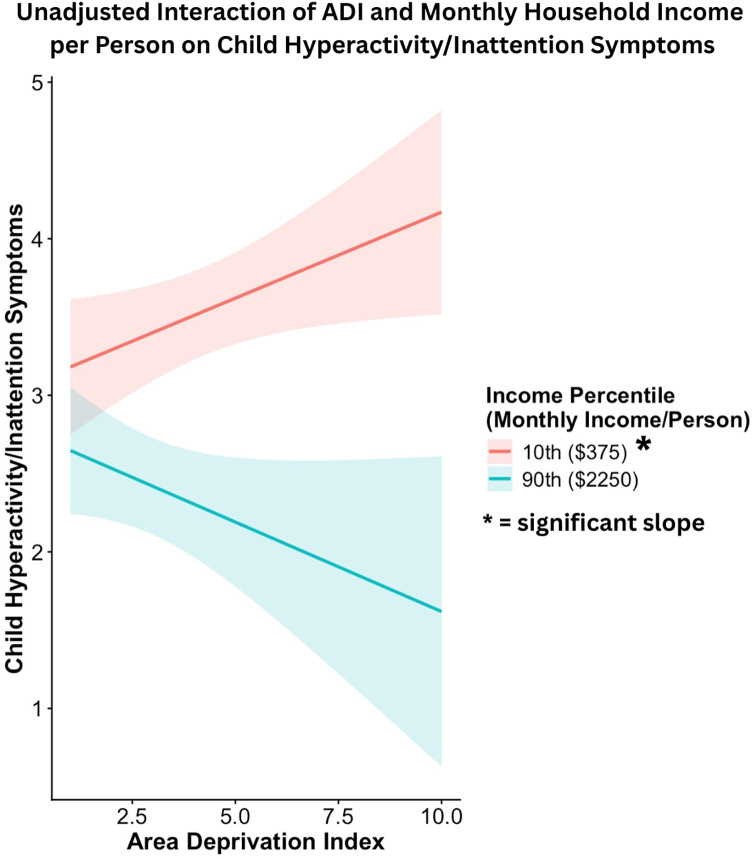
Simple slope analyses illustrating the interaction between ADI state rank and monthly household income in predicting child hyperactivity/impulsive symptoms. Higher prenatal maternal ADI state rank was associated with higher child hyperactivity/impulsive symptoms at low monthly household income per person levels.

We conducted *post hoc* exploratory analyses to examine the association of prenatal maternal ADI state rank and monthly household income per person with the child hyperactivity/inattention subscale of the externalizing problems scale. Generalized linear models showed an interactive effect of prenatal maternal ADI state rank and monthly household income per person on child hyperactivity/inattention scores in the adjusted model (*β* = −0.07 ± 0.032, Wald χ^2^ = 5.06, *p* = .025). As shown in Figures 5 and 6, a higher ADI state rank was associated with higher child hyperactivity symptoms only at lower levels of monthly household income per person. The association between ADI state rank and child hyperactivity symptoms was not significant at average or high levels of income. Significant covariates in this model included child age at assessment (*β* = −0.13 ± 0.032, Wald χ^2^ = 17.53, *p* < .001) and sex (*β* = −0.42 ± 0.068, Wald χ^2^ = 38.62, *p* < .001). Younger child age and male sex were each associated with higher child hyperactivity symptoms. Sensitivity analyses conducted within the Sioux Falls site showed the same pattern of results (see online supplement for details).

### Association of prenatal maternal ADI state rank and monthly household income per person with child conduct problems

We conducted *post hoc* exploratory analyses to examine the association of prenatal maternal ADI state rank and monthly household income per person with the child conduct problems externalizing subscale. Generalized linear models did not demonstrate an interactive effect of prenatal maternal ADI state rank and monthly household income per person on child conduct problems in the adjusted model. We did not observe a significant main effect of prenatal maternal ADI state rank with the child conduct problems subscale in the adjusted model. However, we did observe significant main effects of monthly household income per person (*β* = −0.084 ± 0.034, Wald χ^2^ = 6.16, *p* = .013), age (*β* = −0.16 ± 0.032, Wald χ^2^ = 24.58, *p* < .001), and sex (*β* = −0.33 ± 0.067, Wald χ^2^ = 24.76, *p* < .001) on child conduct problems in the adjusted model. Lower monthly household income per person was associated with increased child conduct problems. Additionally, younger child age and male sex were each associated with higher child conduct problems.

### Association of prenatal maternal ADI state rank and monthly household income per person with child internalizing problems

Generalized linear models did not demonstrate an interactive effect of prenatal maternal ADI state rank and monthly household income per person on child internalizing problems in unadjusted or adjusted models. We did not observe a significant main effect of prenatal maternal ADI state rank with child internalizing problems in the adjusted model. However, we did observe a significant main effect of monthly household income per person (*β* = −0.13 ± 0.036, Wald χ^2^ = 13.28, *p* < .001) on child internalizing problems in the adjusted model. Lower monthly household income per person was associated with increased child internalizing problems.

### Association of prenatal maternal ADI state rank and maternal education with child behavioral outcomes

Generalized linear models did not demonstrate an interactive effect of prenatal maternal ADI state rank and maternal education on any child behavioral outcomes (i.e., externalizing problems, including hyperactivity/inattention scores, conduct problems, and internalizing problems). In models adjusted for sex and age, we observed significant main effects of maternal education on child externalizing problems (*β* = −0.084 ± 0.033, Wald χ^2^ = 6.63, *p* = .01), hyperactivity/inattention scores (*β* = −0.10 ± 0.032, Wald χ^2^ = 8.87, *p* = .0029), and internalizing problems (*β* = −0.08± 0.03, Wald χ^2^ = 6.61, *p* = 0.01). We did not observe a significant main effect of maternal education on child conduct problems in adjusted models. More detailed results of these models, including the main effects of sex and age, are reported in the online supplement.

## Discussion

The current study is the first to examine the interactive effects of prenatal individual-level and neighborhood-level socioeconomic status factors on behavioral outcomes during early and middle childhood. We observed that a higher state-level ADI rank during pregnancy was associated with increased child externalizing problems, but only among families with lower prenatal monthly household income per person. When conducting exploratory *post hoc* analyses on the interactive effects of prenatal neighborhood deprivation and household income per person on child externalizing behavior subscales, we found that only hyperactive and inattentive behaviors were implicated in this association. In contrast, no interactive effect was observed between prenatal ADI state rank and household income per person on child internalizing problems, though significant main effects of household income emerged. Similarly, we found no interactive effects between prenatal ADI state rank and prenatal maternal educational attainment on child externalizing or internalizing problems. However, prenatal maternal education showed significant main effects on child externalizing and internalizing problems. Together, these findings offer a nuanced perspective on how prenatal SES factors relate to children’s behavioral and emotional problems across childhood.

Although the association between individual- and neighborhood-level SES and child behavioral outcomes has been established (Singh & Ghandour, 2012), this is the first study to showcase the interactive effect between prenatal household income and neighborhood deprivation during pregnancy on child behavioral outcomes. While our findings align with extensive research on the impact of SES factors at both the individual and neighborhood level on child behavioral problems, most of this work has not included measures of prenatal SES (Anderson et al., 2022; Barry et al., 2022; de Laat et al., 2016). Notably, we identified that the adverse effects of neighborhood deprivation on child externalizing problems were buffered by higher levels of household income during pregnancy. Our results provide support to recent findings on the potential of income supplementation to new mothers as an effective intervention to improve children’s developmental outcomes (Brownell et al., 2018; Enns et al., 2021; Troller-Renfree et al., 2022).

According to the DOHaD framework, exposure to adverse environments in utero is associated with an increased risk for adverse developmental outcomes (Barker, 1995; Doi et al., 2022; Monk et al., 2019). The present study demonstrated that prenatal exposure to neighborhood deprivation was significantly associated with increased child externalizing behaviors at lower prenatal income levels. Lower prenatal SES has been associated with greater local volumes at the neonatal cerebral surface in regions, including the anterior cingulate gyrus (Spann et al., 2020), which has been associated with greater child susceptibility to maternal parenting behaviors (Nolvi et al., 2020). These findings suggest that children exposed to low prenatal SES may be more sensitive to environmental and neighborhood conditions than those from higher SES backgrounds. The current study extends prior research by demonstrating that prenatal socioeconomic disadvantage is associated with behavioral outcomes in middle childhood (ages 5–13), underscoring the enduring influence of early socioeconomic conditions beyond toddlerhood.

Our findings demonstrate that neighborhood deprivation and monthly household income per person during pregnancy interact to predict child externalizing outcomes. These neighborhood-level effects are supported by literature examining the role of neighborhood adversity on maternal and child health through several pathways. Specifically, parents living in areas with high levels of unemployment, low mean income, and low education levels are more likely to report parenting-related stress than parents living in areas of higher SES (Spijkers et al., 2012; Tung et al., 2024). Prenatal maternal stress has been previously identified as a risk factor for children’s externalizing symptoms (MacKinnon et al., 2017). Evidence from evolutionary psychology suggests that the developing fetus may be sensitive to exposure to stress in utero, which serves to prepare the fetus for a potentially dangerous or unpredictable postnatal environment (Frankenhuis & Del Giudice, 2012). This type of calibration, based on the prenatal environment, has been theorized to predict early individual differences in how young children respond to their environment. Supporting this notion, studies using the ADI and insurance type as SES indicators have found that lower SES at birth is associated with increased externalizing symptoms at 2 years of age (Ramphal et al., 2020).

While child hyperactivity/inattention problems and conduct problems often co-occur and share similar risk factors (Beauchaine & McNulty, 2013; Goodman et al., 2010), the interaction between neighborhood deprivation and monthly household income per person was specific to child hyperactivity/inattention symptoms. Children residing in low neighborhood-level SES are more likely to exhibit hyperactivity/inattention symptomology (Butler et al., 2012), and our findings extend this evidence, which indicates that this effect is particularly pronounced among families with low household income. Consistent with prior work showing associations between ADHD symptoms, SES disadvantage, and neighborhood hardship (Bozinovic et al., 2021; Russell et al., 2014), our findings suggest that higher neighborhood deprivation was associated with heightened child externalizing outcomes only at lower prenatal household income levels per person. Because our analysis did not include potential mediators such as parental involvement (Russell et al., 2014), we cannot infer specific mechanisms.

Unexpectedly, our exploratory analyses revealed no significant interactive effect between neighborhood deprivation and monthly household income on child conduct problems. While our main effects of household income per person align with prior research (D’Onofrio et al., 2009), we did not observe that neighborhood disadvantage independently predicted child conduct problems. In our sample of participants aged 5 to 13 years (*M*age = 8.43), hyperactivity/inattention was more prevalent than conduct problems (see online supplement; Table S2). This result diverges from previous literature (Goodnight et al., 2012), suggesting that the relationship between neighborhood context and child conduct problems may be more complex or duration- and context-dependent than previously assumed (Gutman et al., 2019). Although the current analysis revealed a negative association between child age and higher conduct problems, prior research indicates that conduct disorders tend to emerge during the teenage years, while hyperactivity and attentional difficulties tend to manifest earlier in childhood (Beauchaine & McNulty, 2013; Bennett & Offord, 2001). Furthermore, proposed ontogenic process models of externalizing behaviors suggest that deficits in trait impulsivity serve as a common vulnerability to the sequential development of externalizing spectrum disorders across the lifespan, from temperament (e.g., infant negative affect) to adult externalizing disorders (e.g., substance use disorders) (Beauchaine & McNulty, 2013). Under this model, trait impulsivity is conceptualized as a heritable personality construct that can be shifted towards disorder by adverse exposures (Beauchaine & McNulty, 2013). However, identifying both shared and specific risk factors for child externalizing outcomes is essential for developing targeted interventions, especially considering the resource-intensive nature of programs that address broad social factors such as socioeconomic status (Evans et al., 2025).

Above all, the present study identified how neighborhood deprivation influences child behavioral outcomes, specifically regarding household income per person. Previous research has shown that children from low-income households living in highly deprived neighborhoods face limited access to essential resources like education and childcare, negatively impacting their development (Duncan et al., 1994; Leventhal & Brooks-Gunn, 2000). Neighborhood social infrastructures, such as public libraries, can facilitate interactions between children from different income backgrounds, increasing social capital, and promoting upward mobility (Chetty et al., 2022). These effects often accumulate over time, with family-level SES and neighborhood conditions jointly influencing child outcomes, especially during adolescence (Duncan et al., 1994). The present study’s findings support and extend this literature by highlighting how neighborhood deprivation affects child behavioral outcomes in relation to household income per person.

Our findings are partially supported by the work of Wodtke and colleagues (2016), which showed that adolescent exposure to disadvantaged neighborhoods reduces high school graduation rates for low-income youth. While our study examines prenatal exposure to neighborhood deprivation, rather than adolescent exposure, it similarly suggests that family income moderates the effects of neighborhood-level deprivation on child developmental outcomes. Intervention studies have demonstrated that prenatal income supplements can mitigate socioeconomic disparities in birth outcomes (Brownell et al., 2018; Enns et al., 2021). A recent randomized controlled trial that provided unconditional monthly cash transfers to low-income families in the first year of a child’s life demonstrated higher infant EEG absolute power in mid- to high-frequency bands (Troller-Renfree et al., 2022). Lower EEG power in mid- to high-frequency bands has been observed in other forms of deprivation (Debnath et al., 2020; Marshall et al., 2008) and with externalizing behaviors in both children and adults (Rudo-Hutt, 2015). Additionally, studies have reported that supplying food stamps during pregnancy, as a form of prenatal SES income support, is associated with increased infant birth weight (Almond et al., 2011), crucial for adequate child development. In light of these findings, our study suggests that future research should explore whether the provision of income-based support during the prenatal period could reduce the likelihood of increased externalizing symptoms in children.

## Strengths and limitations

Our analysis’s strengths include leveraging the PASS-ECHO cohort for a large sample size and utilizing an innovative index of area deprivation for a holistic measure of neighborhood-level SES. However, several limitations must be taken into consideration when interpreting our findings. Firstly, the present PASS-ECHO cohort is concentrated within South Dakota, and our population is comprised mostly of White and American Indian or Alaska Native participants. Our South Dakota cohort has a median ADI state rank of 3.0 deciles and a median monthly household income of $3,500, translating to approximately household income of $42,000 per year. This limits the generalizability of our findings to other populations in more urban settings or with different socioeconomic, racial, and ethnic makeup; however, our findings highlight Midwestern rurality as an important dimension of diversity and expand representation of American Indian or Alaska Native communities in research. For researchers interested in examining the relationship between neighborhoods and income on child behavior, it is important to note that the measures used in the present study may need refinement. A New York-based study demonstrated that in regions with significant variations in cost of living, such as New York City, the ADI may be problematic for assessing the level of deprivation, as it relies heavily on the median home value of a neighborhood (Hannan et al., 2023). It is important to note that our sensitivity analyses revealed an interactive effect between ADI state rank and monthly household income per person on child externalizing problems, specifically hyperactivity/inattention, within the Sioux Falls recruitment site (see online supplement for details), suggesting that the results are largely robust to within-site analyses.

By using prenatal maternal ADI and children’s SDQ scores, we were able to assess longitudinal relationships between the prenatal period and child behavioral problems. However, the use of prenatal ADI limited our ability to examine the concurrent effects of neighborhood deprivation and individual-level SES on child behavior postnatally. Recent findings have demonstrated how changes in maternal education and income from the perinatal period to childhood are associated with higher child neurocognitive function (Hackman et al., 2015; Morales et al., 2024), indicating that changes in SES-related factors across development can affect child developmental outcomes. However, because postnatal SES data were unavailable for this analysis, our observed associations may have been confounded by postnatal socioeconomic conditions.

## Conclusion

In the present study, we found that prenatal maternal area deprivation and household income per person had interactive effects on child behavioral outcomes. Notably, our analyses revealed that higher levels of area deprivation and lower levels of monthly household income per person were associated with increased externalizing problems, especially hyperactivity and inattention problems. Additionally, the adjusted models revealed significant main effects of maternal education on both child externalizing and internalizing problems. The present study’s findings suggest that household income per person and neighborhood deprivation interact to impact child externalizing problems. Our findings are consistent with prior research suggesting that income supplementation may be a promising approach to improve birth and child developmental outcomes (Brownell et al., 2018; Enns et al., 2021; Minh et al., 2017; Troller-Renfree et al., 2022). Future studies should explore how changes in individual-level socioeconomic status SES and neighborhood adversity from the prenatal period through childhood influence children’s externalizing and internalizing problems. Such research has the potential to inform public policies aimed at supporting pregnant individuals and promoting positive development across childhood.

## Supporting information

10.1017/S0954579426101151.sm001 Hu et al. supplementary material Hu et al. supplementary material

## Data Availability

The authors have adhered to the Level 2 TOP standards. Select de-identified data from ECHO are available through the Eunice Kennedy Shriver National Institute of Child Health and Human Development’s (NICHD) Data and Specimen Hub (DASH). DASH is a centralized resource, allowing researchers to access data from various studies via a controlled-access mechanism. Researchers will be able to request access by creating a DASH account and submitting a Data Request Form. The NICHD DASH Data Access Committee will review the request and determine whether access is granted in approximately 2–3 weeks. Once granted access, researchers will be able to use the data for 3 years. Information on study data not available on DASH, such as some Indigenous datasets, can be found on the ECHO study DASH webpage. Please see the DASH Tutorial for more detailed information on the request process.

## Supplementary material

The supplementary material for this article can be found at https://doi.org/10.1017/S0954579426101151.

## Pre-registration statement

This work was not preregistered with an analysis plan in an independent and institutional registry.
