# Supplementary material for: Associations of neighborhood deprivation and household income during pregnancy on child externalizing and internalizing problems
Source: Dev Psychopathol. 2026 Feb 6:1–12. Online ahead of print. doi: 10.1017/S0954579426101151 (PMC12888081; doi:10.1017/S0954579426101151)
Supplement: Hu et al. supplementary material [file S0954579426101151sup001.docx]

**SUPPLEMENT**

**CORRELATION BETWEEN DEMOGRAPHIC VARIABLES**

The correlation between demographic variables of sex, monthly household income per person, and education was calculated using point-biserial correlation for binary variables and Spearman correlation for ordinal or continuous variables. Additionally, we found no significant differences in monthly household income per person (p = 0.908), ADI state rank (p = 0.123), and maternal education (p = 0.145) between children who were administered the SDQ once versus children who were administered the SDQ twice. There was a significant difference (p = 0.018) in age between children who were administered the SDQ once (*M*_age_ = 8.53) versus children who were administered the SDQ twice (*M*_age_ = 8.14).

**Table S1.** Means, standard deviations, and correlations with confidence intervals for demographic variables (*n* = 793)

| Variable | *M* | *SD* | 1 | 2 | 3 | 4 |
| --- | --- | --- | --- | --- | --- | --- |
|  |  |  |  |  |  |  |
| 1. Monthly Household Income per Person | 1169.68 | 640.02 |  |  |  |  |
|  |  |  |  |  |  |  |
| 2. Child Female Sex Assigned at Birth | 0.51 | 0.50 | .02 |  |  |  |
|  |  |  | [-.05, .09] |  |  |  |
|  |  |  |  |  |  |  |
| 3. Maternal Educational Attainment | 3.58 | 1.07 | .48** | -.02 |  |  |
|  |  |  | [.43, .53] | [-.09, .05] |  |  |
|  |  |  |  |  |  |  |
| 4. ADI | 3.55 | 2.58 | -.31** | -.02 | -.28** |  |
|  |  |  | [-.37, -.24] | [-.09, .05] | [-.35, -.22] |  |
|  |  |  |  |  |  |  |
| 5. Child’s Age at Administration | 8.43 | 2.17 | .09** | -.02 | .05 | -.02 |
|  |  |  | [.02, .16] | [-.09, .05] | [-.02, .12] | [-.09, .05] |
|  |  |  |  |  |  |  |

*M* and *SD* are used to represent mean and standard deviation, respectively. Values in square brackets indicate the 95% confidence interval for each correlation. The confidence interval is a plausible range of population correlations that could have caused the sample correlation (Cumming, 2014). * indicates *p* < .05. ** indicates *p* < .01.

**SOUTH DAKOTA AREA DEPRIVATION INDEX NATIONAL RANKINGS (2015) HEATMAP**

With the ADI’s 2015 version, South Dakota’s national rankings were visualized using a heatmap to demonstrate the variability of neighborhood deprivation across the United States and highlight the state’s rurality at the national level (Figure S1). The South Dakota cohort has a mean national ADI percentile rank of 49.3 and a median percentile rank of 46.0, with values ranging from the 7th to the 100th percentile.

**Figure S1.** South Dakota Area Deprivation Index National Rankings 2015

**
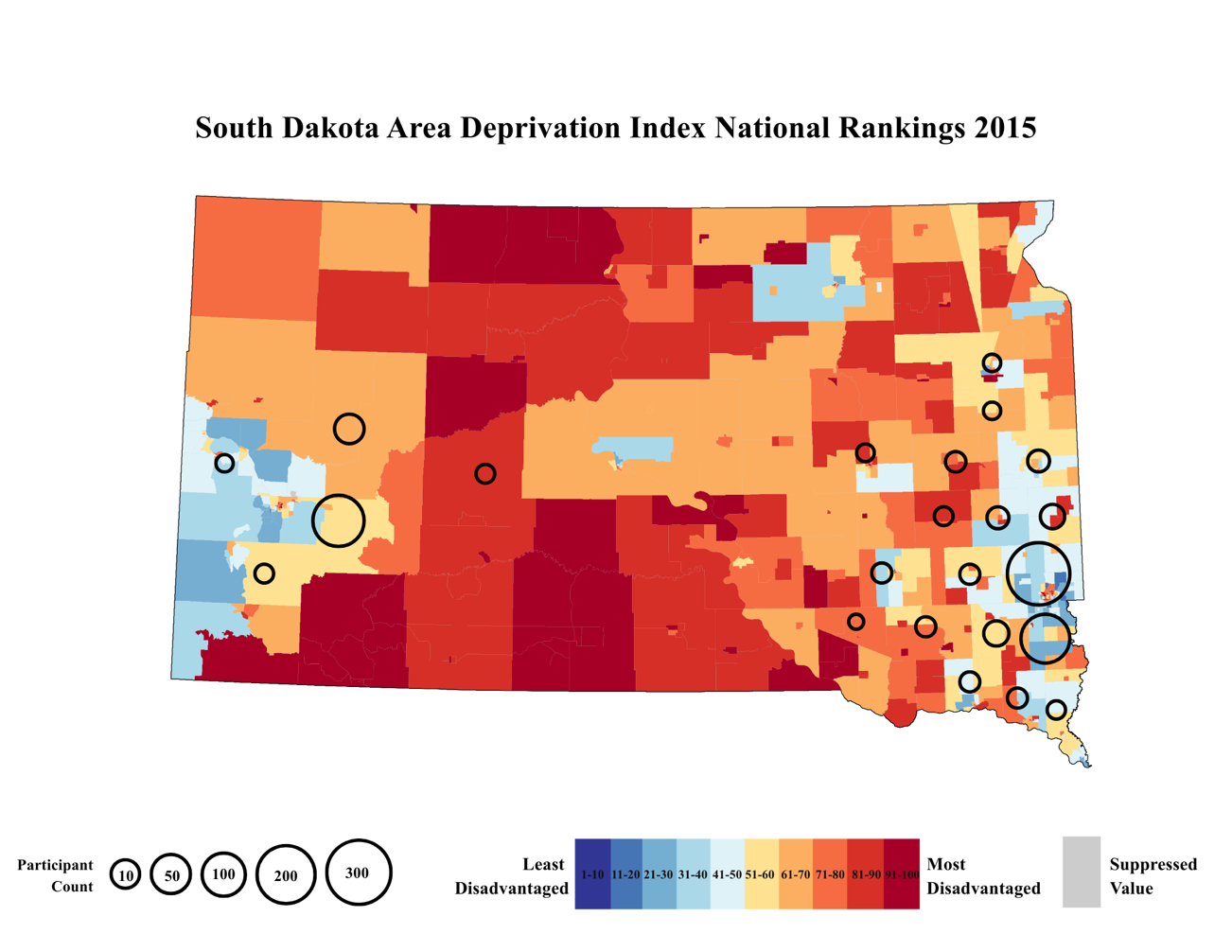
**

**Figure S1.** A heatmap depicting national ADI percentile ranks for South Dakota using the 2015 ADI. Percentile values range from 1 to 100, where lower percentiles (dark blue) reflect lower neighborhood deprivation and higher percentiles (dark red) reflect greater deprivation. Gray regions denote areas with suppressed estimates due to group-quarters population thresholds. Overlaid circles (sizes corresponding to 10–300 participants) indicate participant density across the state (total N = 793).

**DESCRIPTIVE STATISTICS FOR SDQ SCORES**

In the present study, 793 children aged 5 to 13 completed the SDQ. Analyses focused on the externalizing and internalizing scales. Within the externalizing scale, hyperactivity/inattention was more prevalent in our sample than conduct problems.

**Table S2.** Descriptive Statistics for SDQ Scores

|  | **Overall (N=793)** |
| --- | --- |
| **Child Externalizing Problems** |  |
| Mean (SD) | 4.17 (3.33) |
| Median [Min, Max] | 4.00 [0, 18.0] |
| **Child Internalizing Problems** |  |
| Mean (SD) | 2.74 (2.62) |
| Median [Min, Max] | 2.00 [0, 13.0] |
| **Child Hyperactivity/Inattention Problems** |  |
| Mean (SD) | 3.04 (2.40) |
| Median [Min, Max] | 3.00 [0, 10.0] |
| **Child Conduct Problems** |  |
| Mean (SD) | 1.13 (1.40) |
| Median [Min, Max] | 1.00 [0, 8.00] |

**POST HOC ANALYSIS – ASSOCIATION OF PRENATAL MATERNAL ADI STATE RANK AND MATERNAL EDUCATION WITH CHILD BEHAVIORAL OUTCOMES**

**Association of prenatal maternal ADI state rank and maternal education with child externalizing problems**

Generalized linear models did not demonstrate an interactive effect of prenatal maternal ADI state rank and maternal education on child externalizing problems in unadjusted or adjusted models. We did not observe a significant main effect of prenatal maternal ADI state rank on child externalizing problems in the adjusted model. However, we did observe significant main effects of maternal education (β = -0.084 ± 0.033, Wald χ^2^ = 6.63, *p* = .01), age (β = -0.17 ± 0.032, Wald χ^2^ = 28.81, p < .001), and sex (β = -0.46 ± 0.0677, Wald χ^2^ = 45.68, p < .001) on child externalizing problems in the adjusted model. Lower maternal education attainment was associated with increased child externalizing problems. Additionally, younger child age and male sex were each associated with increased child externalizing problems.

**Association of prenatal maternal ADI state rank and maternal education with child hyperactivity/inattention scores**

We conducted *post hoc* analyses to examine the association of prenatal maternal ADI state rank and maternal education with the child hyperactivity/inattention externalizing subscale. Generalized linear models did not demonstrate an interactive effect of prenatal maternal ADI state rank and maternal education on child hyperactivity/inattention symptoms in the adjusted model. We did not observe a significant main effect of prenatal maternal ADI state rank on child hyperactivity/inattention in the adjusted model. However, we did observe significant main effects of maternal education (β = -0.10 ± 0.032, Wald χ^2^ = 8.87, p = .0029), age (β = -0.14 ± 0.032, Wald χ^2^ = 19.89, p < .001), and sex (β = -0.44± 0.068, Wald χ^2^ = 40.78, p < .001) on child hyperactivity/inattention symptoms in the adjusted model. Lower maternal educational attainment was associated with increased child hyperactivity/inattention symptoms. Additionally, younger child age and male sex were each associated with higher hyperactivity/inattention symptoms.

**Association of prenatal maternal ADI state rank and maternal education with child conduct problems**

We conducted *post hoc* analyses to examine the association of prenatal maternal ADI state rank and maternal education with the conduct problems externalizing subscale. Generalized linear models did not demonstrate an interactive effect of prenatal maternal ADI state rank and maternal education on conduct problems in the adjusted model. We did not observe a significant main effect of prenatal maternal ADI state rank or maternal education on child conduct problems in the adjusted model. However, we did observe significant main effects of age (β = -0.16 ± 0.03, Wald χ^2^ = 26.12, p < .001), and sex (β = -0.34 ± 0.07, Wald χ^2^ = 25.80, p < .001) on child conduct problems in the adjusted model. Younger child age and male sex were each associated with higher child conduct problems.

**Association of prenatal maternal ADI state rank and maternal education with child internalizing problems**

Generalized linear models did not demonstrate an interactive effect of prenatal maternal ADI state rank and maternal education on child internalizing problems in unadjusted or adjusted models. We did not observe a significant main effect of prenatal maternal ADI state rank on child internalizing problems in the adjusted model. However, we did observe a significant main effect of maternal education (β = -0.08± 0.03, Wald χ^2^ = 6.61, p = 0.01) on child internalizing problems. Lower maternal educational attainment was associated with increased child internalizing problems.

**Association of ADI state rank and monthly household income per person with child externalizing problems within the Sioux Falls Site**

Within the Sioux Falls recruitment site, generalized linear models showed an interactive effect of ADI state rank and monthly household income per person on child externalizing problems in unadjusted models (β = -0.00018 ± 0.00090, Wald χ^2^ = 4.19, p = .041). In adjusted models, the same interactive effect was a non-significant trend (β = -0.00016 ± 0.00085, Wald χ^2^ = 3.78, p = .052), likely due to the reduction in power (Overall Sample n = 793, Sioux Falls n = 585).

**Figure S2.** Significance of ADI’s effect on child externalizing symptoms by monthly household income per person within the Sioux Falls Site


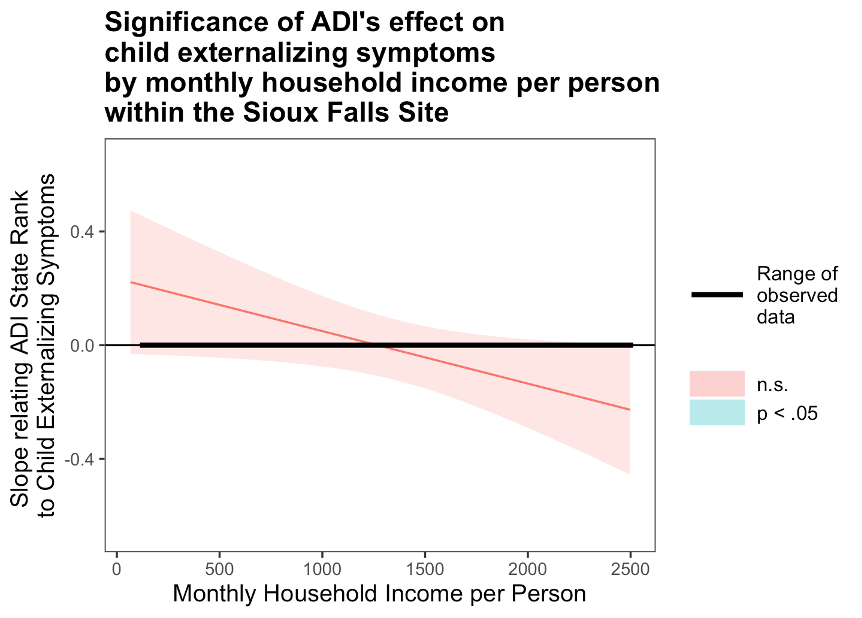


**Figure S2.** Probing unadjusted interactions within the Sioux Falls Site with the Johnson-Neyman technique. The y-axis represents the conditional slope of ADI State Rank as the predictor, while the x-axis represents household income per person in US Dollars. The plot shows where the conditional slope differs significantly from zero. Higher prenatal maternal ADI state rank was associated with higher child externalizing symptoms at low monthly household income per person levels within the Sioux Falls recruitment site, as indicated by the light blue area on the left, where p<0.05.

**Association of ADI state rank and monthly household income per person with child hyperactivity/inattention scores within the Sioux Falls Site**

Within the Sioux Falls recruitment site, generalized linear models showed an interactive effect of ADI state rank and monthly household income per person on child hyperactivity/inattention scores in unadjusted models (β = -0.00015 ± 0.00007, Wald χ^2^ = 4.75, p = .029) and adjusted models (β = -0.00014 ± 0.00007, Wald χ^2^ = 4.30, p = .038).

**Figure S3.** Significance of ADI’s effect on child hyperactivity/inattention symptoms by monthly household income per person within the Sioux Falls Site


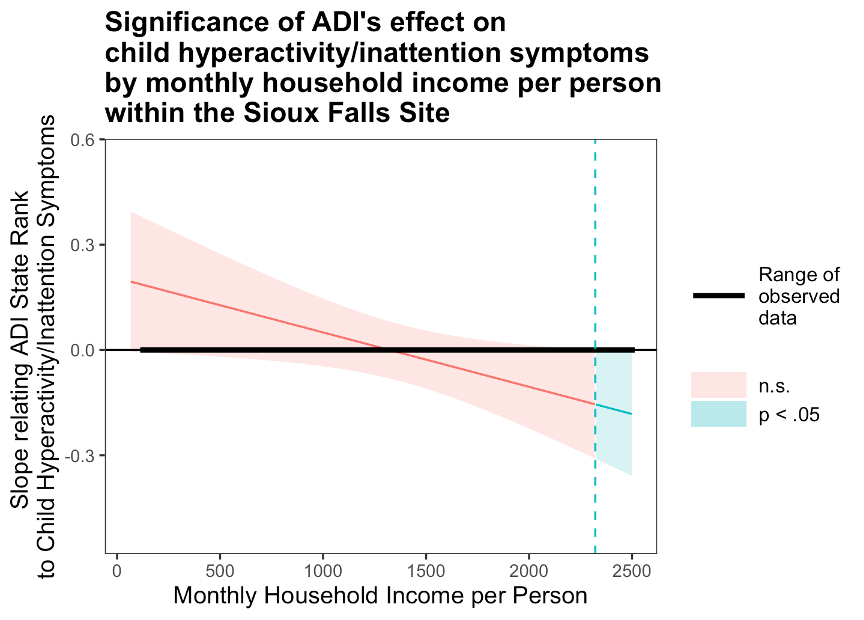


**Figure S3.** Probing unadjusted interactions within the Sioux Falls Site with the Johnson-Neyman technique. The y-axis represents the conditional slope of ADI State Rank as the predictor, while the x-axis represents household income per person in US Dollars. The plot shows where the conditional slope differs significantly from zero. Higher prenatal maternal ADI state rank was associated with higher child hyperactivity/inattention symptoms at low monthly household income per person levels, as indicated by the light blue area on the left, where p<0.05.
